# Biocompatibility and Effectiveness of a Novel, Organic Olive Oil-Based Denture Adhesive: A Multicenter Randomized and Placebo-Controlled Clinical Trial

**DOI:** 10.3390/ijerph18073398

**Published:** 2021-03-25

**Authors:** Luís Azevedo, André Correia, Carlos F. Almeida, Pedro Molinero-Mourelle, Maria Correia, Jaime Del Río Highsmith

**Affiliations:** 1 Department of Conservative Dentistry and Orofacial Prosthodontics, Faculty of Dentistry, Complutense University of Madrid, 28040 Madrid, Spain; lperei02@ucm.es (L.A.); jrh@odon.ucm.es (J.D.R.H.); 2 Centre for Interdisciplinary Research in Health, Faculty of Dental Medicine, Universidade Católica Portuguesa, 3504 Viseu, Portugal; andrecorreia@ucp.pt (A.C.); carloscenfa@gmail.com (C.F.A.); mcorreia@ucp.pt (M.C.); 3 Department of Oral Rehabilitation, Universidade do Porto, 4200 Oporto, Portugal; 4 Department of Reconstructive Dentistry and Gerodontology, School of Dental Medicine, University of Bern, 3010 Bern, Switzerland

**Keywords:** prosthodontics, edentulism, elderly, complete dentures, *Candida albicans*, antimicrobial activity

## Abstract

To assess the clinical efficacy of a novel, organic olive oil-based denture adhesive and its effect on *Candida albicans* growth in maxillary edentulous individuals wearing complete dentures, individuals were selected from two dental schools in Portugal and Spain. Twenty-eight complete dentures were relined, following a standardized protocol. The novel product (test) was compared with a commercialized adhesive (control) and Vaseline (placebo) randomly assigned in a cross-study design. The retention resistance was measured with a gnathometer and a dynamometer. The patients related outcome evaluations with a five-point questionnaire, and the *Candida albicans* growth in a Sabouraud dextrose agar (SDA) medium was used to evaluate differences between the placebo and experimental product. Twenty-three participants were included. The dynamometer evaluation showed significant differences between not using a denture adhesive and using either (experimental, *p* = 0.03; control, *p* = 0.04) and no significant differences between the two adhesives (*p* > 0.05). In the subjective analysis, the experimental adhesive showed a significantly longer effectiveness (*p* = 0.001), and the control reported better results in taste (*p* = 0.03) and in chewing (*p* = 0.001). The test adhesive showed better (*p* < 0.001) *Candida albicans* growth inhibition. The experimental adhesive showed longer effectiveness than the control and the placebo with a better inhibition capacity for the growth of *Candida albicans*. Patients reported better abilities for speech, chewing, taste, and retirement in the control adhesive.

## 1. Introduction

Complete dentures continue to be a reliable treatment option for edentulous patients because of medical and economic constraints [1]. Dentures’ retention, stability, and support depend on the intimate adaptation of their base to the soft and hard tissues, the peripheral seal fir, and saliva’s presence between the dentures and the intraoral tissues [2,3]. However, in several clinical situations, saliva’s natural adhesiveness and the denture base design and extension cannot provide enough stability and retention. In those cases, denture adhesives proved to be a good option for patients by increasing comfort levels and ultimately improving function performance [4,5].

Effective action mechanisms of denture adhesives depend on the combination of physical and chemical forces [6]. When in contact with saliva, denture adhesives increase by 50 to 150% in volume, mainly because of some of their components’ hydrophilic nature. They eliminate the empty spaces between the underlying oral mucosa and the dentures’ base, and they ultimately improve the peripheral sealing and increase the dentures’ stabilization and retention [6].

In the last decades, several studies have been published to determine the effectiveness of denture adhesives [2,7]. Regardless of the various methods used, most of the studies concluded that denture adhesives improve stability and retention and, therefore, contribute to greater user satisfaction and confidence [7,8,9,10,11,12,13,14,15].

Besides retention, another clinical issue that denture wearers often face is oral tissue infection. *Candida albicans* is a commensal organism in the oral cavity of 45–65% of healthy individuals [16,17]. In denture wearers, the prevalence of *Candida* increases from 60 to 100% [16,18]. Several studies have investigated denture adhesives’ effect on oral microbiota but with contradictory results. One study [19] showed that *Candida albicans* growth in vitro was potentiated by some denture adhesives, whereas, in another study [20], denture adhesives showed antifungal behavior [19,20].

Organic oils, including olive oil, have been known for their antioxidant, anti-antioxidant, anti-inflammatory, and antimicrobial properties [21] and have been used in dermatological products for some time [22]. However, proper testing in formulation for oral products has only recently been tested and demonstrated both for caries [23] and surgical and periodontal applications [24].

This clinical study aimed to assess the efficacy of a novel, organic olive oil-based denture adhesive in maxillary edentulous patients wearing complete dentures. Patient-centered outcomes associated with denture adhesive use, such as perceived degree of retention, patient satisfaction, and time of effectiveness were evaluated, as well as the growth of *Candida albicans* in vitro in the presence of both denture adhesives. The null hypothesis was that there were no statistically significant differences between the novel adhesive (test), the conventional adhesive (control), and the placebo adhesive for the clinical outcomes (retention, patient satisfaction, time of effectiveness) and for the *Candida albicans* growth.

## 2. Materials and Methods

### 2.1. Study Design

A randomized controlled clinical trial was designed following The Consolidated Standards of Reporting Trials (CONSORT) guidelines [25] and performed at the Department of Prosthodontics of the Faculty of Dentistry of the Complutense University of Madrid and at the Oral Rehabilitation Unit of the Faculty of Dental Medicine of the Portuguese Catholic University. The protocol was evaluated and approved by the research ethics committee of the Clinic San Carlos Hospital of Madrid, Spain (trial registration code: CEIC 18/515-R_P) following the Declaration of Helsinki’s ethical principles for medical research involving human subjects. Informed consent was obtained from all participants before conducting the research.

### 2.2. Study Participants

The sample size was calculated based on the expected effect for the control adhesive [10], using an alpha value of 0.05 and a statistical power of 80% (G-Power v. 3.1.9.2, Dusseldorf, Germany). Patients were selected from a convenience sample at the Department of Prosthodontics of the Complutense University of Madrid’s Faculty of Dentistry and at the Oral Rehabilitation Unit of the Portuguese Catholic University’s Faculty of Dental Medicine.

Inclusion criteria were as follows: participants older than 18 years, fully edentulous for at least one year, maxillary dentures fabricated within the previous two years, availability for the study appointments, and confirmed absence of allergic sensitivity to the denture adhesive components. Patients with poor oral hygiene and patients incapable of clear communication because of neurological diseases or temporomandibular disorders were excluded from this study.

### 2.3. Study Intervention

The dentures were clinically relined with a hard-reline resin material (Ufi Gel hard C, VOCO, Cuxhaven, Germany) according to the manufacturer’s protocol. The same type of teeth was used for all dentures (SR Orthotyp PE for posterior teeth and SR Vivadent PE for anterior teeth, Ivoclar Vivadent, Lichtenstein) to ensure similar contact/wear. A bilateral balanced occlusal scheme was ensured and verified in all participants. Once relined, dentures were assessed for accuracy and correct adjustment. Participants were asked to wear them for two weeks to allow dentures to adjust and achieve a good fit. After this period, participants entered the experimental part of the study if no mucosa lesion was found.

### 2.4. Experimental Design

An international multicenter crossover, randomized, triple-blinded (the random assignment of denture adhesives and placebo in forms A, B, and C was not known by the investigator, the patient, and the statistical) clinical trial with a 2-week clearance period was adopted. After a 2-week adaptation period with the relined dentures, participants had a baseline-recording visit. During this appointment, initial retention values were registered using a gnathometer and a dynamometer. The initial participant-centered outcomes were also recorded in a questionnaire.

Participants were randomly assigned through Microsoft Excel 2010 software (Microsoft Co., Redmond, WA, USA) for one of the test products. Participants were instructed on the use of the test products (amount, application, and cleaning process) and were asked to use them in the following two weeks.

At the 2-week evaluation visit, the same outcomes measurements were registered, and participants completed another questionnaire. After this visit, participants were instructed to continue using their dentures for the following two weeks without using any product (clearance period). After this period, participants were again asked to use a new assigned product for another two weeks, and similar baseline and 2-week evaluations were made. Once each participant had completed the study using the three products, the visits scheme was repeated.

### 2.5. Study Materials

- Control adhesive (A): conventional denture adhesive, (Kukident Pro^®^, Procter and Gamble, Cincinnati, OH, USA) cream form, composed of Polyvinyl Alcohol-Methyl acrylicate (PVM/MA) copolymers, liquid paraffin, sodium celluloses, petrol, colorings, preservatives, and aromatic particles.
- Experimental denture adhesive (B): novel adhesive, (OlivaFix^®^ Gold, Bonyf AG, Lichtenstein) cream formulated with 30% extra virgin organic olive oil, with no zinc, mineral oil, or Vaseline.
- Placebo adhesive (C): Vaseline (Vaseline, Senti2^®^, Madrid, Spain).

The three tested adhesives were blinded and transferred into identical containers labeled A, B, and C for further assessment. For the clinical evaluation of the dentures’ stability and retention, a disposable gnathometer (Procter and Gamble Co., Cincinnati, OH, USA) and a dynamometer (Correx, Haag-Streit, Bern, Switzerland) were used.

A gnathometer measures the occlusal force required to dislodge a complete denture when both arches occlude simultaneously. The participant bites until the denture moves and the indicator records it on a decimal scale from 0 to 10 units (gnathometer units). When the result of the measurement was between two units the lowest was registered. The measurements were repeated three times, with a one-minute interval, in three locations: the incisors and between the left and right first molars. The mean value of these measurements was used for analysis.

A dynamometer quantitatively measures (grams (g)) the force necessary to dislodge the denture from the residual ridge of the patient when traction is applied. Three records were made first in the anterior area of the frenulum and then in the posterior lateral areas. The mean value of these measurements was used for the analysis.

### 2.6. Outcome Variables

The main assessed variables were the dentures’ retention and stability, measured using the gnathometer and the dynamometer, as described above. The participant-centered outcomes were recorded using a questionnaire. This questionnaire evaluated, on a scale of five items (very good, good, moderate, minimal, or very bad), the participant’s subjective evaluation of the following variables: retention and stability, taste and consistency of the denture adhesive, denture adhesive intra-oral removal capacity, and the participant’s willing to use the denture adhesive again. The clinical protocol and the questionnaire were adapted from Pradíes et al. [10].

### 2.7. Pilot Study–Inter- and Intra-Observer Reliability

A pilot clinical study was performed to determine the intra-observer measurement variability. Five participants with complete maxillary dentures were selected from each faculty. Participants had a natural dentition, a fixed prosthesis, or a removable prosthesis (on teeth or implants) in the antagonist arch. For each participant, the primary evaluator made six measurements (three with the dynamometer and three with the gnathometer only in the anterior area) with a one-minute break. One week later, at the same period of the day, the measurements were repeated for each participant with the same gnathometer. The measurement deviation was calculated using the Dahlberg formula, and the Pearson correlation was calculated as the reliability coefficient.

Another pilot study was carried out in the Faculty of Dental Medicine clinic of the Portuguese Catholic University to determine the interobserver concordance. Two main evaluators used the same gnathometer and dynamometer to obtain the corresponding measurements in all participants. They made three measurements in the anterior area, and the average value was used in the analysis. All measurements were made after a one-minute break to allow the participant to restore the denture comfortably. A three-way analysis of variance (ANOVA) was applied to assess the observers’ influence due to the possible learning effect, while correcting patient results. The measurement error was calculated by the Dahlberg formula, and the concordance coefficient was calculated by the Pearson correlation.

### 2.8. Effect of Denture Adhesives on Candida Albicans Growth

In vitro evaluation of the effect of denture adhesives on the growth of *Candida albicans* ATCC 11225 was evaluated on solid Sabouraud dextrose media, following protocols similar to those used by Sampaio-Maia et al. [17]. Saturated solutions of the denture adhesives (1% *w*/*v*) were prepared in sterile saline solution (0.9% NaCl) and added to a sterile Sabouraud dextrose agar (SDA). Thereafter, each plate (20 mL) of SDA received 1 mL of denture adhesive solution and was inoculated in triplicate with 0.5 mL of a standardized inoculum of 1 × 10^6^ cells/mL of *Candida albicans* ATCC11225 and incubated at 25 °C for a week. The plates were observed in three moments, at 48, 120, and 168 h, and the colony forming units (CFUs) were counted.

### 2.9. Statistically Data Analysis

Collected data were transferred to a database (Microsoft Excel, Microsoft, Redmond, WA, USA) and analyzed by two independent researchers. Subsequently, data were statistically processed with SPSS (SPSS for Windows, version 25, SPSS Inc., Chicago, IL, USA). For each test, normality was verified with Kolmogorov–Smirnov and Shapiro–Wilk statistical tests. For quantitative questions, the Kruskal–Wallis test was used to analyze possible differences between the test products in the absence of normality. In case of discrepancies, the Mann–Whitney test was applied to each product’s results separately and the Bonferroni correction to a combination of the two. When normality was verified, an ANOVA was used, as well as the post-hoc Bonferroni test. The qualitative questions were expressed in percentages and with a chi-square test.

Results of the in vitro analysis were expressed in CFU/milliliter, corresponding to cells/milliliter. All measurements were obtained in triplicate, and all tests were repeated once. Means were calculated, and results were compared with a Student’s *t*-test or with an ANOVA analysis. For all tests, α = 0.05 was used, and *p*-values equal to or less than 0.05 (*p* ≤ 0.05) were considered statistically significant.

## 3. Results

### 3.1. In Vivo Assessment

**Figure 1:**
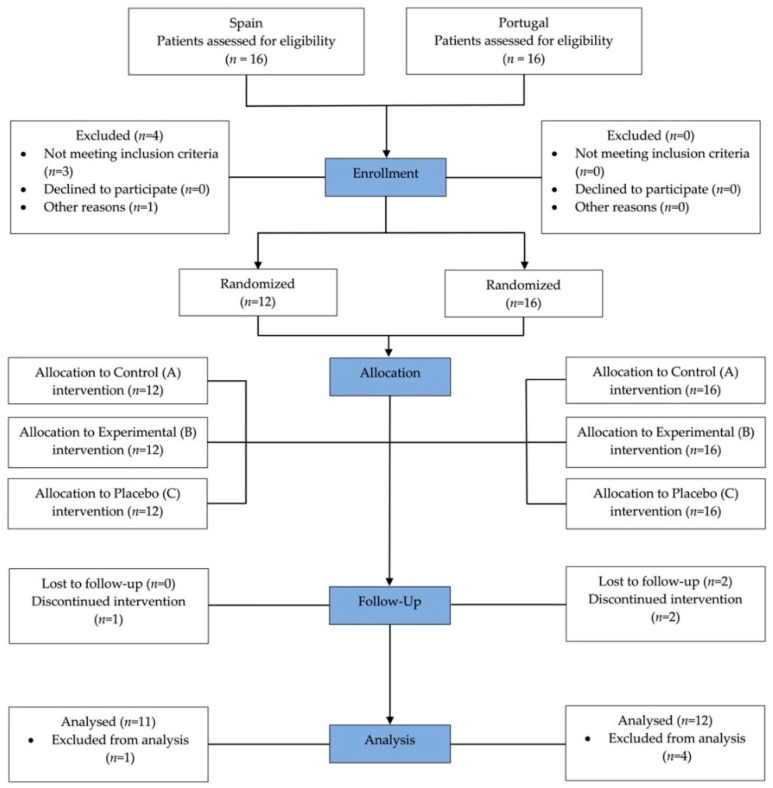
Flowchart diagram of the study protocol according to The Consolidated Standards of Reporting Trials (CONSORT) guidelines [25].

Thirty-two patients were recruited to participate in this study and 28 participants were finally included: 12 (42.86%) in the Complutense University of Madrid and 16 (57.14%) in the Universidade Católica Portuguesa. Five (17.86%) individuals did not complete the experimental part of the study and were not considered in the evaluation. The remaining participants (*n* = 23) completed the study (Figure 1).

Most of the participants were older than 65 years (y) old (*n* = 15; 65.20%; (45–89); mean: 68 y) and female (*n* = 15; 65.22%). The average age of their current dentures was 1.57 years, with a range between one (*n* = 10; 43.5%) and two years (*n* = 13; 56.5%). Regarding the frequency of denture’s hygiene, although the clear majority of individuals did clean their denture at least twice a day (*n* = 15; 65.2%), 30.4% (*n* = 7) did it only once a day, and 4.3% (*n* = 1) did not clean them. The 69.6% (*n* = 16) of the sample reported having already used denture adhesives before, however, 62.5% (*n* = 10) had stopped using them, reporting a bad taste (*n* = 2; 8.7%) and ineffectiveness (*n* = 4; 17.4%) as the main causes.

Based on the questionnaire responses, the participant-centered outcomes reported that the experimental denture adhesive had longer effectiveness, which, for 73.91% (*n* = 17) of the sample, was greater than 8 h (*p* = 0.0001). The control adhesive presented better results, improving the speaking (*p* = 0.003) and chewing ability (*p* = 0.001) and facilitating the removal/cleaning of the adhesive from the denture (*p* = 0.003), with statistically significant differences (Table 1 and Table 2).

There were no statistically significant differences between the three products in the subjective evaluation of the dentures’ retention and stability. The overall intra-observer measurement error was 0.15 gnathometer units, whereas the overall interobserver measurement error was 0.12 gnathometer units. The intra-observer reliability coefficient was 0.90, whereas the overall interobserver reliability coefficient was 0.60 g for the dynamometer and 0.80 g for the gnathometer. The ANOVA showed no systematic observer effect in the evaluations with the dynamometer (*p* = 0.178) and the gnathometer (*p* = 0.78).

According to Table 3, the mean force needed to dislodge the dentures, measured with a dynamometer, was about 155.8 ± 51.5 g when the experimental denture adhesive was used and was 152.7 ± 52.6 g with the control denture adhesive. Although the force required to dislodge the dentures with the experimental denture adhesive was more significant than that required with the control denture adhesive, that difference was not statistically significant (*p* > 0.05). The results of the gnathometer measurements (Table 3) revealed that when the experimental denture adhesive was used the necessary force was about 1.0 ± 0.6 units and when the control denture adhesive was used it was 1.1 ± 0.6. There were no statistically significant differences between the three groups (*p* = 0.055).

### 3.2. In Vitro Assessment

**Figure 2:**
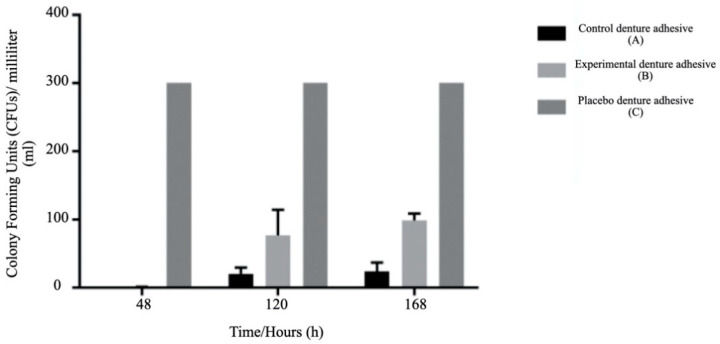
Effect of the denture adhesives on the growth of *Candida albicans* ATCC 11225 in the Sabouraud dextrose agar (SDA) medium.

The effect of the denture adhesives on the SDA is shown in Figure 2; after 48 h, there was no growth of *Candida albicans* ATCC 11225 in the presence of the denture adhesives. However, after five days, there was growth with both. The experimental adhesive had a higher impact on inhibiting the growth of *Candida albicans* ATCC 11225 by showing a significant difference (*p* < 0.05) in the number of CFUs five and seven days after the test compared to the control adhesive and the control (*Candida albicans* with no product). Although there was some growth of *Candida albicans* ATCC 11225 in the presence of the two denture adhesives (experimental and control), the growth was slower and less intense with the experimental denture adhesive.

## 4. Discussion

The null hypothesis was rejected since significant differences were found between the assessed adhesives. The present study selected dentures with a maximum wearing period of two years according to Maeda et al. [26]; a denture needs to be relined after approximately 27 months. The ideal protocol would be the evaluation of new dentures, as in other studies [10,27]. Nevertheless, considering the short evaluation period of our research, and the fact that dentures require an adaptation period, a bias could occur. Therefore, we decided that the best method would be to fit and reline the dentures with a hard reline, and then verify that they all had a correct bilateral, balanced occlusion. The present study was a phase IV clinical trial in which a new product (OlivaFix^®^ Gold) was evaluated and compared with a previously studied product (Kukident Pro^®^) and a placebo (Vaseline^®^). Vaseline was selected as the placebo because of its consistency, similar to denture adhesives, and its lack of a specific flavor.

This study was designed as a multicenter to evaluate these products more efficiently by obtaining a good sample of participants to satisfy the study’s objective within a reasonable time frame. A pre-team meeting was held to standardize the procedures as much as possible, and a pilot study was conducted to standardize and calibrate the clinical evaluators. All procedures were recorded in video format in case there were doubts.

Regarding the influence of the products on chewing improvement, the control denture adhesive presented better results (*p* = 0.001) than the experimental denture adhesive. These denture adhesives were significantly better than the placebo (*p* = 0.001) for both. These results agree with several studies [14,28,29], which found that denture adhesives led to an increase in the chewing rate and a decrease in the duration of chewing cycles. Although the differences between the two denture adhesives were significant, in descriptive terms, the number of participants who stated that their chewing improved with the control denture adhesive was not much higher than those for the experimental denture adhesive (*n* = 16; 69.57% vs. *n* = 13; 56.52%).

In terms of effectiveness time, the experimental denture adhesive had significantly longer effectiveness than the control (*p* < 0.0001). Its composition may explain these results. The experimental adhesive features an innovative formula using a high concentration of olive oil instead of commonly used ingredients, such as zinc and petrolatum. Scientific evidence indicates that denture adhesive’s retention force decreases over time [28] because of its dissolution [30]. As explained above, when the adhesive is in contact with saliva, it slowly absorbs water and increases in volume, increasing in viscosity until the hydrophilic polymer particles come into contact with each other to form a continuous polymer matrix. Subsequently, oral fluids destroy the polymer matrix, decreasing the viscosity and resulting in progressively weaker bond strength. Therefore, the olive oil, which is highly viscous, in the experimental denture adhesive composition, may explain the significantly longer adhesion effectiveness.

In this research, two quantitative variables were combined. First, by using a dynamometer, we tried to simulate participants’ force when speaking, smiling, and doing other daily activities. Then, with the gnathometer, the objective was to simulate movements that occur during chewing. Thus, the combination of these evaluation methods allowed us to test a wide variety of movements that can influence the stability of complete dentures. However, it should be noticed that the evaluated forces do not consider the frequency, duration, and magnitude of the functional or parafunctional forces carried out on a daily routine. The average value of the tensile force required to dislocate the denture when using the denture adhesives was approximately 150 g. These results are lower than those reported in other studies [10,31], which ranged from 350 g to 1095 g. However, Pradíes et al. [10] evaluated new dentures, which may justify that observation. Nonetheless, if we analyze the scientific evidence regarding implant-retained overdentures, retention values were found between approximately 350 and 500 N, according to the different retention systems used [32,33]. Thus, the values obtained with denture adhesives would not be those expected in the overdentures with ball retention systems, for example.

Finally, regarding the gnathometer values, in general, the results indicated similar mean values for the two denture adhesives. Additionally, the reported values with the use of denture adhesives were higher than those obtained without these products. However, according to inferential analysis, there were no significant differences between the three products. Other authors obtained average values much higher than ours [2,11,27]. On the other hand, the results obtained by Pradíes et al. [10], with and without denture adhesives, are in agreement with ours. If we do not consider units and focus only on the percentage of increase or improvement with the use of denture adhesives compared to the use of none (37.5%), our study also agrees with that of Polyzois et al. [11] (32.5%) on the evaluation of some denture adhesives.

When interpreting these results, it should be borne in mind that most of the studies, except for the study by Pradíes et al. [10], only obtained measurements from the anterior zone. In contrast, in our study, the evaluations were performed in three locations (two posterior and one anterior), and the average value was calculated; this may justify the discrepancies obtained.

Regarding the inhibition of *Candida albicans* growth, although there was a fungal growth in the presence of the two denture adhesives (experimental and control), the experimental denture adhesive had a greater effect on inhibiting that growth, with statistically significant differences at days five and seven of the trial. We could expect better results with the control denture adhesive because it has zinc, which is known to have antifungal activity [34]. However, the experimental denture adhesive, which has no zinc in its composition, showed a more significant antifungal effect. This effect may be due to the organic olive oil in the experimental denture adhesive, which is known to have phenolic components that have anti-inflammatory and even anti-cancer properties [35]. Dacrory et al. evaluated the use of olive oil by-products in a new antimicrobial hydrogel [35]. They discovered that this product has an antimicrobial capacity against *Staphylococcus aureus*, *Pseudomonas aeruginosa*, and *Candida albicans* [36]. Therefore, although the experimental denture adhesive does not contain zinc (a classic antimicrobial) in its composition, the presence of these possible by-products of the organic olive oil may be responsible for the antifungal effect it had in this study.

A limitation of the laboratory evaluation is that in vitro observations are not always representative of the in vivo situation. Saliva components and salivary flow and variable intraoral pH can interfere with the growth of *Candida albicans*. Furthermore, since there is an association between oral *streptococci* and *Candida albicans* [37], it would also be essential to test the denture adhesives’ effect on the growth of oral *streptococci*.

Further multicenter, international studies are needed to assess the differences and similarities between countries regarding denture adhesives. In addition, educational programs for patients regarding this issue should be developed. Feedback and comments from participants can be valuable to the manufacturers of these products, thus improving several less successful aspects. It would also be important to develop guidelines or protocols regarding denture adhesives.

## 5. Conclusions

Within the limitations previously discussed, the following conclusions can be drawn:

- There were no differences in the force required to dislodge the denture under traction between the experimental and the control denture adhesives.
- Differences in individuals’ evaluation of the dentures’ retention and stability were not statistically significant among the three products.
- The experimental adhesive showed a better effectiveness time than the control and placebo.
- The control denture adhesive improved the ability to speak and chew, taste and odor, and ease of removal with significant differences.
- The experimental denture adhesive showed the best antimycotic effect against the growth of *Candida albicans* compared to the control and placebo.

## Figures and Tables

**Table 1:** Descriptive analysis of the patient-centered outcomes.

Participant-Centered Outcomes Concerning Denture Adhesives	Speaking Improvement		Chewing Ability		Satisfaction Degree				Taste		Effectiveness Time				Opinion on Utility			Cleaning/Removal	
	Yes	No	Yes	No	No	Little	Sufficient	Very	Yes	No	Less than 4 h	4–8 h	8–12 h	More than 12 h	Zero	Little	Effective	Easy	Difficult
Control denture adhesive (A)	78.26% (18)	21.74% (5)	69.57% (16)	30.43% (7)	13.04% (3)	21.74% (5)	56.52% (13)	13.04% (3)	34.78% (8)	39.13% (9)	60.87% (14)	8.70% (2)	47.83% (11)	34.78% (8)	8.70% (2)	13.04% (3)	21.74% (5)	73.91% (17)	26.09% (6)
Experimental denture adhesive (B)	65.22% (15)	34.78% (8)	56.52% (13)	43.48% (10)	8.70% (2)	47.83% (11)	39.13% (9)	8.70% (2)	91.30% (21)	56.52% (13)	43.48% (10)	8.70% (2)	17.39% (4)	73.91% (17)	0.00% (0)	8.70% (2)	47.83% (11)	39.13% (9)	65.22% (15)
Placebo denture adhesive (C)	26.98% (6)	73.91% (17)	21.74% (5)	78.26% (18)	21.74% (5)	52.17% (12)	26.09% (6)	21.74% (5)	60.87% (14)	39.13% (9)	39.13% (9)	78.26% (18)	13.04% (3)	8.70% (2)	0.00% (0)	21.74% (5)	52.17% (12)	26.09% (6)	8.70% (2)

**Table 2:** Inferential analysis of the patient-centered outcomes.

Participant-Centered Outcomes Concerning Denture Adhesives	Speaking Improvement	Chewing Ability	Satisfaction Degree	Taste	Effectiveness Time	Cleaning/Removal
	*p*	*p*	*p*	*p*	*p*	*p*
Control denture adhesive (A) vs placebo denture adhesive (C)	**0.004**	**0.001**	**0.024**	**0.009**	**0.001**	0.01
Control denture adhesive (A) vs experimental denture adhesive (B)	**0.003**	**0.001**	0.073	**0.027**	**0.001**	**0.026**
Experimental denture adhesive (B) vs placebo denture adhesive (C)	**0.004**	**0.001**	0.09	0.365	**0.001**	**0.037**

*p* ≤ 0.05.

**Table 3:** Dynamometer and gnathometer measurements.

Groups	Dynamometer		Gnathometer	
	Mean ± SD (g)	*p*-Value	Mean ± SD	*p*-Value
No denture adhesive	123.8 ± 38.3	vs (B)—**0.034** vs (A)—**0.041** vs (C)—1.000	0.8 ± 0.6	0.055
Control denture adhesive (A)	152.7 ± 52.6	vs initial—**0.041** vs (B)—0.995 vs (C)—**0.048**	1.1 ± 0.6	
Experimental denture adhesive (B)	155.8 ± 51.5	vs initial—**0.034** vs (A)—0.995 vs (C)—**0.047**	1 ± 0.6	
Placebo denture adhesive (C)	122.7 ± 36.0	vs initial—1.000 vs (B)—**0.047** vs (A)—**0.048**	0.8 ± 0.5	

## Data Availability

The data that support the findings of this study are available from the corresponding author, P.M.-M., upon reasonable request.
